# A win-win scenario? Restrictive policies from alternative standpoints

**DOI:** 10.1108/JHOM-06-2021-0239

**Published:** 2021-11-22

**Authors:** Caroline Cupit, Natalie Armstrong

**Affiliations:** Department of Health Sciences , University of Leicester , Leicester, UK

**Keywords:** Health services, Healthcare, Public sector management, Qualitative research, Quality improvement, Social sciences

## Abstract

**Purpose:**

In this viewpoint article, the authors consider the challenges in implementing restrictive policies, with a particular focus on how these policies are experienced, in practice, from alternative standpoints.

**Design/methodology/approach:**

The authors draw on social science studies of decommissioning work to highlight how patient and official versions of value often vary, creating difficulties and distrust as restrictive policies are implemented. Patients and the public are well aware that financial calculations are somehow embedded in concepts of “evidence” and “value” but are usually unfamiliar with the social infrastructures that produce and utilise such concepts. The authors discuss with reference to a contemporary restrictive programme in England.

**Findings:**

While policymakers and researchers frequently present restrictive policies as “win-win” scenarios (achieving both cost-savings for healthcare services and improved patient care), social science analyses highlight the potential for tensions and controversies between stakeholders. The authors recognise that cost containment is a necessary component of policymaking work but argue that policymakers and researchers should seek to map (and make visible) the socially organised reasoning, systems and processes that are involved in enacting restrictive policies. Although transparency may pose challenges, it is important for informed democratic engagement, allowing legitimate scrutiny of whose voices are being heard and interests served (the “winners” and “losers”).

**Originality/value:**

The authors argue for social science analyses that explore overuse, value and restrictive practices from alternative (e.g. patient) standpoints. These can provide important insights to help identify priorities for intervention and support better communication.

## Introduction

The “overuse” of medical services is increasingly recognised as an international problem ( Organisation for Economic Co-operation and Development, 2017 ). Defined as “the provision of medical services that are more likely to cause harm than good” ( Brownlee *et al.* , 2017 ), it is understood as one form of “inappropriate care” (along with underuse and misuse) and frequently employed contiguously with the more economically focussed concept of “low-value care” ( Porter, 2010 ).

Initiatives to identify and address overused interventions have proliferated, with many aiming to improve patient/practitioner understandings of the relative benefits and harms of intervention, and promote shared decision-making in which patients “choose less” ( Schpero, 2014 ). More recently, researchers have increasingly argued for system-level approaches that use financial levers within reimbursement systems to limit the options available within clinical decision-making ( Pathirana *et al.* , 2017 ).

In England, contemporary examples of system-level initiatives to address overuse include the *Evidence* *-* *Based Interventions (EBI) programme* ( NHS England, 2020 ). Co-ordinated nationally, EBI guidance to local Clinical Commissioning Groups ( NHS England *et al.* , 2018 ) mandates them to restrict the provision of interventions that are listed as inappropriate, using financial levers to ensure compliance. The list initially comprised 17 interventions which “should not be routinely commissioned” or which “should only be commissioned or performed when specific criteria are met”, with plans in place for extension ( “Evidence-based interventions”, 2020 ). These interventions are accompanied by guidance on items that should no longer be routinely prescribed in primary care ( NHS England, 2019a ). The EBI programme sits alongside many other cost-saving initiatives that are conceived and implemented by regional policymakers and which utilise national and local sources of evidence pertaining to value.

Policymakers typically present restrictive policies as politically neutral processes for improving “value” – reducing patient harm and system costs. However, controversies around the EBI programme, for example, highlight that some stakeholders (notably patients and practitioners advocating for them) understand restrictive policies differently. Although the programme's main objective is presented as clinical improvement (with any cost savings being reinvested in patient care ( NHS England, 2019b )), critics have argued that it is fundamentally geared towards the delivery of cost savings ( Keep Our NHS Public, 2019 ) and that it will be *detrimental* to patient care ( Keep Our NHS Public, 2019 ; Madsen, 2018 ; Wathen, 2018 ).

In this article, we trouble policymakers' representations of restrictive programmes as uncomplicated improvement in “value” – when this is presented as an objective, scientific and politically neutral concept. Although sometimes there may be clear-cut opportunities to both stop harmful or ineffective practice *and* make cost savings, these are reasonably rare. More often difficult decisions have to be made, and decision-making processes inevitably create “winners” and “losers”. Here, we spotlight the work of policymakers who enact restrictive policies. In doing so, we shed light on the tensions that arise as official representations of evidence and value bump up against alternative patient understandings of what is valuable in different circumstances. In our discussion, we argue that policymakers and social researchers should deliberately capture understandings of value from a patient standpoint and open up to scrutiny the socially organised systems and processes involved in the implementation of restrictive policies.

## Troubling official representations of evidence and value

Contrary to representations in policy, the production of evidence about overuse is not an entirely scientific or neutral enterprise but rather is a social process with multiple political, economic and relational dimensions ( Knaapen, 2013 ; Morden *et al.* , 2014 ; Cupit *et al.* , 2021 , 2022 ). As Elshaug *et al.* (2012) pointed out in their early report of 150 potentially low-value practices, “services that are ineffective and/or unsafe across the entire patient population to which they are applied are probably quite rare”. Instead, overuse occurs along a continuum, running from “universal benefit” to “entirely ineffective” ( Brownlee *et al.* , 2017 ), and is often established due to an “absence of evidence” rather than “evidence of absence” ( Garner *et al.* , 2013 ). The “easy hits” – i.e. those interventions with a relatively uncontentious evidence base to demonstrate they are “entirely ineffective” for distinct groups of patients – were the first to be addressed ( Robinson *et al.* , 2012a , b ), leaving others in the “grey zone” to be tackled ( Brownlee *et al.* , 2017 ).

In the “grey zone”, interventions that have *negligible or no benefit to patients* are more difficult to establish unequivocally. Not only that, but concepts of evidence and value that are used in identifying services for system-level restriction are complicated by health-economic calculations, of “health outcomes achieved per dollar spent” ( Porter, 2010 ). Policymakers are tasked with patching together various measures of clinical benefit from diverse research studies, with calculations of relative return on investment. This patching together – involving social processes described by Morden *et al.* (2014) – is carried out within a healthcare system underpinned by what is easily measurable (e.g. from clinical trials and economic calculations) and politically expedient ( O'Mahony, 2018 ). Processes for evaluating evidence and determining value underpin who is determined to be eligible to which services at local level. Consequently, the evidence base for restriction has become subject to considerable debate, with definitions, underlying principles and interests being contested in the research literature and by stakeholders.

## Making decommissioning work visible: managing tensions between official accounts and stakeholder perceptions

To date, problems of “overuse”, and the systems designed to address it, have received little sociological attention ( Armstrong, 2021 ). It is only through studies of decommissioning “on the ground” that we see where tensions arise, and how they play out in practice. A small number of existing social science analyses of (de)commissioning (within the English healthcare system) highlight that this work takes place within “a realm of *practice* as well as of ideas, interests and institutions” ( Freeman *et al.* , 2011 ) [emphasis added]. In the case of the EBI programme, the restriction of the specified interventions is based on national NICE guidance and is mandatory for local policymakers. But it does not follow that change is simple to achieve. In practice, local policymaking practice is based on trust and relationships with established provider organisations and involves a range of partnerships and time-consuming micro-processes – which are not as linear or technocratic as implied by the terms “knowledge translation” or “getting evidence into practice”. Indeed, Wye *et al.* (2015) found that the so-called “evidence based policymaking” was (in practice) a pragmatic exercise involving the “selection of ‘evidence’ such as best practice guidance, clinicians' and users' views of services and innovations from elsewhere” and that “local data often trumped national or research-based evidence” – with the utilisation of disparate (and often poor quality) evidence from variation-modelling, benchmarking exercises, analyses of high-volume procedures or localised evidence reviews. Within local policymaking, national policies such as the EBI programme exist as one strand of work within a complex array of other cost containment activities.

In the local settings in which services are configured, policymakers work with ideas of “evidence” and “value” to achieve decommissioning (and other) goals. In this practical context, tensions have arisen between official accounts of value and the perceptions of stakeholders; some restricted (“low-value”) interventions appear to clinicians and patients to be valuable in some circumstances ( Robinson *et al.* , 2012a , b ). Such tensions are accentuated when restrictive policies are mandated, leaving little room for clinical judgement and requiring practitioners and patients to engage in complex processes to determine eligibility and/or exceptionality.

Arising from these tensions has been a cascade of activity, with policymakers becoming embroiled in negotiation and debate with providers, and managers/practitioners manipulating restrictive policies – for example, “gaming” the electronic record system in order to access treatment on behalf of their patients or “making a lot of noise” in news media ( Hollingworth *et al.* , 2015 , p. 78). Both policymakers and providers have been drawn into labour-intensive work to audit activity (i.e. the number and type of procedures carried out, how these are electronically coded) and review contracts and payment mechanisms ( Hollingworth *et al.* , 2015 ). Studies of independent funding request (IFR) processes (which are put in place as a mechanism for determining “exceptionality” alongside restrictive policies) have highlighted the huge amount of representational (interpretive and rhetorical) work that is sometimes required from multiple stakeholders (patients, clinicians and policymakers) in order to make a case for treatment ( Russell *et al.* , 2014 ). Patients without the necessary emotional or financial resources have been less able to contest funding decisions themselves or to present themselves in a favourable way to clinicians who can advocate on their behalf ( Owen-Smith *et al.* , 2009 ).

Policymakers have responded to tensions between different stakeholder concepts of value by employing three distinct approaches. First, they have made extensive discursive efforts to portray their work as “reducing waste” rather than “rationing” ( Rooshenas *et al.* , 2015 ). Second, they have kept restrictive policies and other disinvestment initiatives under the public radar by allowing services to “wither on the vine” or extending waiting lists ( Williams *et al.* , 2017 ; Daniels *et al.* , 2013 ). Third, they have choreographed Patient and Public Involvement exercises, in which patients and the public *appear* to have a voice but do not have (and are not given) the knowledge, opportunity or power to influence strategic decision-making ( O'Shea *et al.* , 2019 ).

## Taking alternative standpoints to track how restrictive policies work in practice

A core part of healthcare commissioning and management is to determine which services and treatments should be funded, and who should have access to them. As treatment options proliferate (new treatments arise and others become redundant), it is essential from a healthcare management perspective to ensure good value from the services provided – and intervene where this is not being achieved. In a tax payer or insurer-funded system, achieving some definition of good value is also likely to be important to patients and the public. However, as we have shown, the practical processes for utilising “evidence” and determining “value” are complex – with the very concepts of “evidence” and “value” being contested by stakeholders in practice.

Social science studies of (de)commissioning and other cost-containment processes are useful in highlighting the (often very resource-intensive) work involved, the difficulties experienced by diverse stakeholder groups and the systems and processes that exacerbate these difficulties. Research that elucidates *patients' experiences* of restrictive policies is particularly important, as the so-called “low-value” care may actually be valuable to patients (and the practitioners advocating for them) in the context of their own unique health needs. Many of these “low-value” services are also relatively low- *cost* services that some patients will be willing and able to fund privately (e.g. Vitamin D/painkiller purchases, varicose vein treatment, cataract removal). The restriction of such services is likely to have a disproportionate impact on the health of disadvantaged groups – not only due to restricted access but also because short-term improvements to everyday well-being may be particularly important for people in these groups. We also observe (e.g. from teaching exercises with medical students) that such “low-value” treatments (for which it is often difficult to demonstrate quantified outcomes and which may be perceived as trivial) are less likely to garner support in public consultation.

We make the case for more research on overuse and restrictive policies that arises from a patient standpoint (and/or from a variety of different patient, practitioner and other marginalised standpoints) ( Cupit *et al.* , 2021 , 2022 ). This kind of research is largely absent from the evidence base. Research from a patient standpoint would explore what patients with a variety of conditions, and in different life circumstances, value from their care – and how they prioritise intervention. It would make visible the difficulties (the practical activities and challenges) that are involved for patients who are impacted by restrictive policies. New studies should focus on groups less able to advocate for themselves, whose conditions are under-represented in research and/or which attract less effective advocacy arising from commercial interests ( McCoy *et al.* , 2017 ) – e.g. the elderly, minority ethnic groups, people with complex, stigmatised or invisible conditions.

As we have highlighted, social science research on cost-containment within policymaking practice is limited. In addition to research on patient experiences, greater understanding is needed about how restrictive policies such as EBI guidance and other initiatives are formulated through various committee-based systems and processes and then implemented *in everyday (de)commissioning practice* . Good quality ethnographic studies in particular can cross the boundaries of national and local organisations to empirically map how such restrictive work is being socially organised within the “multiple and overlapping spaces” of policymaking and healthcare delivery ( Freeman *et al.* , 2011 ) – and *actively produces* problematic experiences for policymakers, practitioners, patients and the public ( Griffith and Smith, 2014 ). Such work might elucidate, for example, the tensions arising between different stakeholder understandings of “value”; how the treatment of some conditions may become “soft targets” for disinvestment; how patients understand and navigate restrictive policies; whose interests are privileged or downplayed across the healthcare system and the impact on (in)equalities. Ultimately, a more nuanced account can be produced that will go beyond simplistic narratives of “improving value” and support future policy in this area.

We do not dispute that difficult decisions need to be made about resource allocation within finite resources – and believe that patients understand this. However, the small number of social science analyses we have referenced point to a lack of transparency over the underpinning assumptions and processes involved in the implementation of restrictive policies – which, in turn, creates distrust between stakeholders. This is unfortunate because many system-level initiatives rely on practitioner and patient support for their implementation. For example, the EBI programme's developers envision that shared decision-making between doctors and patients will be “a key mechanism to support implementation of the programme” ( Markham, 2019 ) – in other words, that patients will align with restrictive efforts based on “evidence” and “value”, when these are explained in frontline care.

We are arguing that the socially organised processes involved in designing and implementing restrictive policies should be opened up to scrutiny, considering how the activities and perspectives of different stakeholders intersect. In particular, policymakers and researchers should better recognise and explore the tensions that belie policymakers' notions of “win-win scenarios”. Although greater transparency about the everyday systems and processes for managing overuse may be perceived as politically risky, social science analyses can provide an important foundation for understanding the consequences for different stakeholder groups and improving communication about the difficult decisions that have to be made.
